# Candida-Associated Denture Stomatitis and Murine Models: What Is the Importance and Scientific Evidence?

**DOI:** 10.3390/jof6020070

**Published:** 2020-05-23

**Authors:** Carolina Yoshi Campos Sugio, Amanda Aparecida Maia Neves Garcia, Thaís Albach, Gustavo Simão Moraes, Estevam Augusto Bonfante, Vanessa Migliorini Urban, Karin Hermana Neppelenbroek

**Affiliations:** 1 Department of Prosthodontics and Periodontics, Bauru School of Dentistry, University of São Paulo, Alameda Octávio Pinheiro Brisolla, 9-75, Bauru, SP 17012-901, Brazil; carolina.yoshi@hotmail.com (C.Y.C.S.); amanda_maia1994@hotmail.com (A.A.M.N.G.); estevamab@gmail.com (E.A.B.); 2 Department of Dentistry, State University of Ponta Grossa, Avenida General Carlos Cavalcanti, 4748, Uvaranas, Ponta Grossa, PR 84030-900, Brazil; thais.albach@gmail.com (T.A.); moraes.gustavo29@yahoo.com.br (G.S.M.); vanurban@yahoo.com (V.M.U.)

**Keywords:** *Candida*, complete denture, murine stomatitis model, review

## Abstract

Considering the high prevalence and recurrence of *Candida*-associated denture stomatitis (CADS), in vivo studies in animal models are necessary before those in humans to evaluate new therapeutic strategies. This study aimed to review the literature on murine models of CADS induction using acrylic intraoral devices simulating dentures. Rats are recommended as experimental animals in these models as well as the adoption of a pasty diet. For maintenance in the proper position during the experiments, intraoral appliances must be obtained by individual impressions, using and retained exclusively by cementation on the molars. The region of interest for histopathological analysis was standardized as that corresponding to the area between the first molars. However, there is no consensus among the studies on the CADS induction rat models in relation to the *Candida albicans* inoculation and need for immunosuppression and/or administration of antibacterial drugs of animals. The greatest difficulty of the available models refers to maintaining the course of the lesion for a sufficient period to evaluate the effectiveness of the proposed treatment, considering the rapid and efficient murine immune response to candidal colonization. Therefore, future studies are necessary for the development of a robust and reproducible CADS model.

## 1. Introduction

Edentulism is still considered one of the main oral health problems since it affects a large part of the world population. The use of complete dentures is a widely indicated option, especially for its low cost. Therefore, special attention is needed for those who use mainly superior removable dentures, as they favor *Candida* colonization by providing a propitious environment for their growth with low PH, low oxygen concentration, and a reduction of local salivary flow. These factors are worsened when the complete dentures are poorly adapted or oral hygiene is failing [1].

Oral candidosis is an opportunistic infection caused by overgrowth and penetration of the oral tissues by pathogenic forms (hyphae and pseudohyphae) of *Candida* spp., mainly *Candida albicans* [1]. *Candida*-associated denture stomatitis (CADS), the most frequent type of oral candidosis and the most common mucosal alteration in the elderly, affects approximately 65% of removable denture wearers and has been associated with multiple etiologic factors [2]. Despite its etiology involving systemic and local factors, CADS is mainly associated with an overgrowth of *Candida* of pathogenic form on the denture surface as a biofilm. The first crucial step of denture biofilm formation is adherence of yeast-form cells to the acrylic surfaces. This process relies on several cell wall proteins, called adhesins, that promote the attachment to other cells (both epithelial and microbial cells), and denture surfaces by binding to specific amino acid or sugar residues. After attachment, the colonization phase begins, in which cell proliferation begins, forming a basal layer of anchoring cells. The maturation of this biofilm occurs in sequence, including the growth of pathogenic fungi form concomitant with the production of extracellular matrix material. At least, yeast-form cells are dispersed from the biofilm to seed new sites [2]. Mucosal infections, including CADS, involve biofilm formation, usually including the interaction with commensal bacterial flora and a host component. Pathogenic forms of *Candida* present in the denture biofilm give the fungus the properties of adhering and invading the denture-bearing mucosa, resulting in infection [1]. 

Typical palate lesion of CADS clinically characterized by reddened spots, diffuse homogeneous erythema, or areas with changes in palatal mucosa texture [3]. In immunocompromised patients, with uncontrolled diabetes, HIV, nutritional deficits, or organ transplants candidosis is difficult to treat, and recurrence is very frequent [4,5,6,7]. Untreated disease in these patients at risk can progress to candidemia, a highly lethal invasive infection with mortality rates beyond 60% [8]. Several alternatives have been studied for the CADS treatment: antifungal therapy, both systemic and topical application [9,10,11,12,13]; disinfectants and cleansing agents [13,14,15]; laser treatment of palatal tissue [16,17]; oral hygiene instructions [13]; denture removal at night [12]; microwave disinfection [10,12]; denture relining procedures [13]; replacement of the old denture [18]; and combined approaches [13]. 

Antifungal therapy, mainly with topical agents, has been established as a conventional treatment for CADS. However, transient improvement, high recurrence, and fungal resistance have been observed [19]. Although systemic antifungal agents are recommended for immunocompromised patients, it is necessary to consider the possibility that the pathology is the result of an endogenous infection [11], besides the potential hepatotoxic and nephrotoxic effects of these drugs, and the interaction with other medications [20]. Although it is still the most used treatment for CADS, topical antifungal therapy with agents such as miconazole, and especially with nystatin [10,12,20] has limitations. Such agents have a short retention time on denture surfaces and infected tissues due to salivary flow, tongue movements and swallowing [10]. The progressive re-infection of the oral mucosa and internal denture surface by *Candida* spp., commonly observed in the short and long-term after discontinuing systemic and topical antifungal therapies [10,11,12], has been attributed to several factors besides the potential problem of the emergence and selection of yeast strains resistant to these drugs [21]. They are unable to maintain in a therapeutic concentration on the inner denture surfaces, resulting in rapid candidal recolonization [13]. Moreover, the dosage of antifungal agents is strict, requiring patient compliance, which may be limiting for the elderly [22]. The action of antifungal drugs conventionally used for the CADS treatment also becomes reduced in denture bases due to the microbial colonization in depth in acrylic resin and the complex biofilm present in this substrate [23]. Two consequences of biofilm growth with great clinical relevance are the lesser susceptibility of microbial cells to the action of antimicrobial agents [19] and greater protection of microorganisms to the action of host defense cells [24]. Low growth, altered regulation of cell metabolism due to the limitation of nutrients and stress conditions and cell density are other suggested mechanisms of biofilm resistance to antimicrobials. This degree of resistance increases with the biofilm maturation [24] and even the species that produce it have been correlated with the unfavorable results in patients with *Candida* infections, including recurrences [8]. Finally, it is important to consider the contact between injured tissues and denture biofilm is not eliminated with the conventional antifungal therapies, thus favoring reinfection of the mucosa through the denture [13].

Although replacing dentures may be a viable therapy for these disadvantages, it has not been effective in eliminating the pathogenic forms of *C. albicans*, which reflects the need to first treat mucosal infection [18]. This may be achieved by denture relining procedures, especially with soft materials modified by antifungal drugs [25]. In addition to the comfort promoted by these products, the sustained-release drug delivery system by this protocol breaks the reinfection cycle, aids the mucosal recovery and reestablishes the adaptation of the denture base to supporting, thus reducing the necessity of patient compliance [22]. However, the scientific evidence for this protocol is restricted to in vitro laboratory tests [15,22,25,26].

In vitro studies do not address the factors that favor biofilm infection, as they are not exposed to immune salivary components and proteins that can coat the surface and promote adhesion. There are also limitations due to the absence of additional components that likely impact the fungal growth state, such as bacterial flora, contact between the device and the host biofilm, dynamics, interference from salivary immune components [27]. Each one of these situations makes this interaction very complex and results in more reliable outcomes in studies developed in vivo [27], which proves the effectiveness and residual responses of the therapy proposed prior to human studies. In this way, the in vivo evaluation of the effectiveness of these new treatment protocols, materials, and drugs is hampered by the lack of an appropriate model to induce CADS in animals.

Investigation of oral candidosis using animals has often been carried out in murine models, which are developed in small rodent animals like mice, rats and hamsters [28,29,30,31,32,33,34,35,36,37,38]. These animals have demonstrated colonization patterns and lesions of oral candidosis similar to those observed in humans both microscopically and histologically [39,40]. Despite that, there remains no consensus in these models regarding a method that induces and maintains the infection for a sufficient period to evaluate the effectiveness of new materials and drugs. Considering this need, the high prevalence of CADS in the dental clinic and the challenges inherent to its treatment due to the high relapse rate after the suspension of conventional antifungal therapy, the present study aimed to review the investigations on murine models of CADS induction with the perspective of determining the most reliable and reproducible method that allows the evaluation of effective therapies for this pathology.

## 2. The Difficulty of Induction of CADS in Animals

The rodent animals of choice for a model murine of CADS induction are rats and not mice, since the former have a cavity compatible with the use of acrylic intraoral devices simulating dentures [27,39,41,42,43,44,45]. Moreover, the clinical and histologic findings in the rats’ palatal tissues of experimental CADS are similar to those of humans [39]. Despite these advantages, a literature search on induction of this lesion in rat models revealed only a few related studies [28,29,30,31,32,33,34] since most previous investigations on murine oral candidosis used mice as experimental animals with lesions induced on the dorsum of the animal’s tongue [28,29,30,31,32,33,34,35,36,37,38]. Therefore, these latter studies can be considered only indirectly since mice have a small oral cavity for the use of palatal denture-like appliances, which are essential for CADS induction [28,29,30,31,32,33,34,35,36,37,38]. 

There are numerous oral candidosis induction protocols available in the literature; however, there is a great divergence between methodologies. Some researchers have reported the induction of oral candidosis by inoculating the fungus directly into the oral cavity of the animals, without inducing a CADS itself [16,17], while others used intraoral devices to develop the lesion [27,39,41,42,43,44,45,46,47,48,49], or even the combination of both methods [43,49]. In the studies using intraoral appliances for inducing CADS in rats [27,39,41,42,43,44,45,46,47,48,49], there is a lack of standardization in their manufacturing, which can make reproducibility difficult and result in maladjusted devices with poor contact with the palatal supporting tissues of the animals [42]. 

After oral candidosis induction, the evaluation of lesion longevity is impaired in murine models due to the efficient and rapid immunological response of the animals to various changes [50]. They have a high ability to resolve infections spontaneously and in a short period; this includes not only *Candida* infections but also more severe lesions, such as induced carcinoma [51,52]. Since most rats do not have *C. albicans* as a commensal fungus, resistance to infection is attributed to their innate immunity induced in the oral cavity [53]. This response can often eliminate the fungus within a few days, making it difficult to obtain a reliable murine model for efficacy treatment against the induced pathology. This difficulty may explain the lack of evaluation on the lesion longevity in the scarce studies available on CADS induction [27,39,41,42,43,44,45,46,47,48]. 

## 3. Problems for *Candida* Inoculation

Oral fungal inoculation is the procedure of rubbing a swab with *Candida* inoculum on the mucosal surface of the animals. In most studies, *Candida* inoculation is done on the dorsum of the animal’s tongue [16,17,29,31,38,54], which has a tissue more susceptible to fungal colonization since its epithelium is not pronounced, dense, or keratinized. Additionally, unlike on the palate, the presence of papillae in the tongue gives it characteristics of a smooth mucosa [40]. However, in order to simulate clinical conditions as previously described, CADS must be induced by *Candida* inoculation on the palate and in denture-like appliances [27,39,41,42,43,44,45,46,47,48,49]. Another aspect that demonstrates the importance of assessing the palatal mucosa under the intraoral devices is the similarity of the histological findings of animals in the CADS induction models with those observed in human lesions such as the presence of intense and diffuse inflammatory infiltrate in the epithelium, microcess areas [27,39,41,42,43,44,45,46,48,49], parakeratosis [39], mitotic activity [43], increased neutrophils in the tissue [45] and the presence of hyphae [27,41,44,46].

*C. albicans* is considered the most reported pathogen in the development of CADS [10]. This fungus has the ability to adhere to both the denture supporting mucosa and acrylic resin bases. The surface roughness and porosity of acrylic resins are relevant factors in the entrapment of microorganisms, mainly *C. albicans*. Its adhesion to mucosal surfaces is related to the virulence of the fungus, chemical and structural characteristics of the cell wall, such a morphological transition between yeast and hyphal forms, the expression of adhesins and invasions on the cell surface, thigmotropism, the formation of biofilms, phenotypic switching and the secretion of hydrolytic enzyme [55]. For these reasons, *C. albicans* is the species of choice for palatal and device inoculation in CADS induction studies [27,39,41,42,43,44,45,46,47,48,49].

Some studies of murine oral candidosis have used immunosuppression and/or antibacterial drugs therapy to reduce the animals’ immune response to infection as well as to favor *Candida* colonization [38,39,42,45,56].

Administration of antibacterial drugs to the animals in murine models prior to inoculation is indicated to reduce the number of competing oral bacteria, allowing yeast overgrowth and resulting in overt fungal infection [38,40,56]. Antibacterial drugs, particularly tetracycline, is often associated with the oral candidosis development in humans [40]. It has also been shown that this specific antibacterial drug was able to affect the size of the lesions caused by *C. albicans* inoculation, although it did not result in significant differences in the number of infected animals [57]. Moreover, tetracycline proved to be effective in causing earlier and more intense oral candidosis lesions in rats [45], in addition to being recommended in combination with an immunosuppressant for the pathology induction in murine models due to the expected increase in fungal colonization [54,58].

Immunosuppression is performed by corticosteroids [27,29,31,54] that can be administered before or on the day of fungal inoculation. These drugs have been previously used in combination with antibacterial drugs, commonly tetracycline [38,59] and this protocol was recommended to provide an appropriate CADS model for further evaluation of various therapeutic agents or treatment approaches. On the other hand, precaution should be taken when adopting immunosuppression due to the damage caused to the animals, since it disrupts activity and causes weight loss and often death. Additionally, there is no agreement with this procedure because CADS does not only affect immunocompromised patients [60]. Lastly, some CADS induction studies have used healthy animals (without immunosuppression), which allowed the participation of their immune response during colonization [39,42,45], as there is no scientific evidence that this pathology is associated with an invasive infection in humans.

Diet is also an important factor in keeping the intraoral devices in the mouth throughout the study period, as well as to limit the accumulation of food debris between the appliances and the palate. It has been recommended to provide a pasty or liquid diet before (for the adaptation of animals) and during the study instead of solid food [27,42,61,62]. 

## 4. Current Scenario and Future Perspectives

The use of intraoral devices simulating dentures is essential in murine models of CADS induction to mimic how the pathology occurs clinically, as these prostheses are one of the most important facilitators for its development [39], besides other local factors, such as the presence of denture biofilm or local trauma caused by the appliances, especially when they are poorly adapted and continually worn [1]. Nevertheless, the literature shows a great divergence in the manufacture of these devices.

For intraoral appliances, previous investigations have used denture base acrylic resin with a steel band around the upper incisors and fixed with self-curing resin [41], fixed and removable portion structures with magnets [42], orthodontic wire fixation [27], palate-coated acrylic resin appliances and with molars fixed with composite resin [63], among others. However, some disadvantages such as premature device loss and a small size variability between devices have been observed by the authors. The fact that intraoral devices need to be used makes the methodology more complex, which requires prior training of the researcher. In addition, it is important that the animal’s weight and daily activity are monitored throughout the process to ensure animal welfare and prevent possible loss.

Due to the evident lack of standardization for this purpose, the study by Hotta et al. (2017) [61] developed a standardized and useful experimental animal model with an intraoral device that may undergo relining, for utilization in future in vivo studies. The authors concluded that the best conditions for achievement of an intraoral device that remains adequately adapted in the oral cavity during the period of treatment CADS with conventional topical antifungal drugs (14 days) were obtained with the following parameters in rats: individual impression, cement-retained intraoral devices, histological specimens of soft/hard tissue, and the region of interest for histopathological analysis corresponding to the area between the first molars, from one neurovascular bundle to the other [61]. This rat model proved to be easily reproducible since it has already been successfully tested in a previous study [62]. Despite these favorable results with the adjustment of palatal intraoral devices, it is important to emphasize that CADS induction was not evaluated by Hotta et al. [61,62]. On the other hand, there was no standardization of oral devices for such parameters in previous investigations of CADS induction animal models [27,39,41,42,43,44,45,46,47,48,49].

The few studies available on rat models for CADS induction are still limited in relation to the development of a lesion for a period long enough to evaluate new therapies and drugs. As soon as the intraoral devices are removed, rats are euthanized for histopathological analysis, thus limiting the evaluation of the long-term effectiveness of treatment to conclude whether lesion remission was due to the treatment protocol or the animal’s immune capacity [27,39,41,42,43,44,45,46,47,48]. Therefore, further studies are needed to establish an effective protocol to favor colonization by *C. albicans* (antibacterial therapy and/or immunosuppression) as well as clinical microbiological, and histopathological parameters for determining the longevity of infection in the tested animals. 

## 5. Conclusions

Based on the findings of this review, the following conclusions were drawn:

1. In the animal models of CADS induction, there is a consensus in the pertinent literature on the use of rat as an experimental animal, strains of *C. albicans* for fungal inoculation, intraoral devices simulating acrylic dentures for the development of lesions, and a pasty diet to prevent the detachment of appliances;
2. For the maintenance in proper position and correct adaptation in the mouth during the experiments, the intraoral devices must be obtained by individual impression, being exclusively retained by cementation on the molar region;
3. Regarding the histological parameters of the support tissue evaluation area, specimens of soft/hard tissues are recommended, as is a region of interest for histopathological analysis corresponding to the area between the first molars;
4. There is still a lack of consensus regarding the development of a reproducible, accessible, and reliable murine model for the induction of CADS in which the infection remains for a sufficient period to evaluate the effectiveness of new therapeutic protocols. So, further research is needed to establish the need to induce immunosuppression, as well as whether or not to use antibacterial drugs in rats prior to *C. albicans* inoculation in intraoral devices;
5. It is also essential to determine by clinical, microbiological and histopathological parameters, the extension of the permanence of the acrylic devices into the oral cavity of animals before treatment initiation to allow an adequate evaluation of the therapeutic effect, considering the rapid and efficient murine immune response to *C. albicans* colonization.
